# Emotion Measurements Through the Touch of Materials Surfaces

**DOI:** 10.3389/fnhum.2019.00455

**Published:** 2020-01-17

**Authors:** Cyril Bertheaux, Rosario Toscano, Roland Fortunier, Jean-Christophe Roux, David Charier, Céline Borg

**Affiliations:** ^1^Université de Lyon, ENISE, LTDS, UMR 5513 CNRS, Saint-Étienne, France; ^2^ISAE-ENSMA, Chasseneuil-du-Poitou, France; ^3^University Hospital of Saint-Étienne, SNA-EPI Laboratory, EA 4607, CHU, Université de Lyon, Saint-Priest-en-Jarez, France; ^4^University Hospital of Saint-Étienne, CMRR Neuropsychology, Department of Neurology, Université de Lyon, Saint-Priest-en-Jarez, France

**Keywords:** touch, emotion, material, dilation of the pupil, explicit and implicit measures

## Abstract

The emotion generated by the touch of materials is studied via a protocol based on blind assessment of various stimuli. The human emotional reaction felt toward a material is estimated through (i) explicit measurements, using a questionnaire collecting valence and intensity, and (ii) implicit measurements of the activity of the autonomic nervous system, via a pupillometry equipment. A panel of 25 university students (13 women, 12 men), aged from 18 to 27, tested blind twelve materials such as polymers, sandpapers, wood, velvet and fur, randomly ordered. After measuring the initial pupil diameter, taken as a reference, its variation during the tactile exploration was recorded. After each touch, the participants were asked to quantify the emotional value of the material. The results show that the pupil size variation follows the emotional intensity. It is significantly larger during the touch of materials considered as pleasant or unpleasant, than with the touch of neutral materials. Moreover, after a time period of about 0.5 s following the stimulus, the results reveal significant differences between pleasant and unpleasant stimuli, as well as differences according to gender, i.e., higher pupil dilatation of women than men. These results suggest (i) that the autonomic nervous system is initially sensitive to high arousing stimulation, and (ii) that, after a certain period, the pupil size changes according to the cognitive interest induced and the emotional regulation adopted. This research shows the interest of the emotional characterization of materials for product design.

## Introduction

The touch is an essential sense to the human because it allows the contact with our environment, the perception of wind, humidity, temperature changes, relief, roughness, softness, grip (Barnett, 1972). The touch is the most developed sense at birth (Field, 2001). Various studies have shown the influence of massage on brain development and improvement of visual function in premature rats, primates and humans (Stack and Muir, 1990; Field, 1998; Kaffman and Meaney, 2007; Guzzetta et al., 2009), with effects after the first week of stimulation in puppies (Sale et al., 2004). The lack of caresses is known to be a cause of death in orphaned babies (Spitz, 1945) and in baby monkeys (Harlow and Harlow, 1962). Study on maternal care of baby rats established that adult rats descended from a mother who gave numerous licks/groomings showed lower levels of fear reactivity than rats descended from a mother who dispensed very little (Menard et al., 2004; Dunbar, 2010). The pleasant properties of caresses on cats, dogs, humans have often been demonstrated (Harlow, 1959; De Waal, 1989; Lindgren et al., 2010). The therapeutic properties of touch are well known, and it seems that the touch has a significant impact on the neuro-hormonal system (Bush, 2001). Different hormonal changes have been observed in tactile stimulations: change in the rate of cortizol in children after a simple contact with the skin of their mother, change of insulin levels with significance effects on type 2 diabetes, secretion of oxytocin during a pleasant touch (Feldman et al., 2010; Baldini et al., 2013; Clermont, 2018). Human skin is considered as a social organ (Löken and Olausson, 2010). Recent studies suggest (i) that the degree of allowed touch can predict the closeness of a relationship (Suvilehto et al., 2015), and (ii) that the degree of actual touch can predict functional connectivity of the social brain (Brauer et al., 2016). Other studies on social relation showed that a caress activates different emotional cerebral areas, depending on whether participants believe it to come from a person of the same or of the opposite sex (Dolinski, 2010; Gazzola et al., 2012).

The psychophysical dimensions of touch have also been studied (Taylor and Lederman, 1975; Jones and Lederman, 2006; McGlone and Reilly, 2010; Zahouani et al., 2013; Abdouni et al., 2017, 2018). The “epicritical” sensitivity corresponds to the fine and discriminative touch (shape, contours, texture, and relief) (Mountcastle, 2005), while the “protopathic” sensitivity corresponds to the cutaneous sensitivity triggered by strong stimulation, like temperature. The “protopathic” sensitivity generates a defense reaction of the body (Melzack and Wall, 1965).

The “epicritical touch” is achieved by Meissner’s corpuscles and Merkel’s receptors. Meissner’s corpuscles are speed detectors that encode motion. Merkel’s receptors are sensitive to intensity, pressure and temporal information on stimuli. The “protopathic touch” is achieved by Pacini’s corpuscles and Ruffini’s receptors. Pacini’s corpuscles are sensitive to vibrations and record temporal information on stimuli. Ruffini’s receptors are sensitive to the stretching of the skin. The skin also includes thermoreceptors, nociceptors and itch receptors that are sensitive to thermal sensations, pain and skin irritation and itching (Nordin, 1990; Misery and Ständer, 2010; Misery, 2014). Touch also involves proprioceptors, which are sensitive to changes of position and angular velocity of an articulation, tendon. There are three main types of proprioceptors: free nerve endings, Golgi receptors and Pacini corpuscles (Hasan, 1992; Edin, 2001).

Okamoto et al. (2012) have identified five dimensions of the “epicritical” touch that affect our tactile perceptions: hardness *(hard, soft)*, temperature *(hot, cold)*, friction properties *(wet, dry, sticky, slippery)*, fine roughness *(rough, smooth)* and macro roughness *(uneven, relief).* Lederman and Klatzky (1987) have defined the gesture adapted to the tactile characterization of objects and surfaces. Gibson (1962) and Wall (2000) propose to distinguish the “Active” or voluntary touch, and the “Passive” or involuntary touch. During an “Active” touch, the mechanoreceptors of human skin treat tactile, proprioceptive and kinesthetic information which are relayed to the central nervous system to produce the emotional and sensory quality of the material. Some studies have focused on the study of perceptive sensory or emotional lexicons (Melzack, 1975; Essick et al., 1999, 2010; Cardello et al., 2003; Guest et al., 2011). Other studies have investigated the links between physical properties of materials and associated hedonic sensations (Chen et al., 2009: Datta, 2016). These different studies offer a better understanding of the emotional aspects of materials in order to master their emotional impact in a sensory design process.

The aim of this paper is to investigate pupil size variation in response to touch of different materials. It is the first study relating emotional touch with pupillometry. After describing the link between touch and emotional development, the experimental procedure is detailed, and the results are discussed.

## Touch and Emotional Development

Studies on emotional touch have advanced through the study of a patient (GL) with Guillain-Barré syndrome (Forget and Lamarre, 1987). This patient had lost almost all large myelinated afferents, resulting in a total deficit of discriminative touch, over the whole of the body. Despite this deficit, she was able to describe the movement of a soft brush on her arm, as a light, pleasant and gentle touch. A little before, Zotterman (1939) had identified non-myelinated C-Tactile fibers (CT) in cats. These fibers have also been found in the hairy skin of mammals (Leem et al., 1993; Li and Ginty, 2014; Liljencrantz and Olausson, 2014; Rutlin et al., 2014; Walsh et al., 2015; Moayedi et al., 2016). The qualitative feelings of affective stimuli are comparable in hairy and glabrous skin. Recent studies suggest that different CT fibers have been found in the glabrous skin and have different biochemical and structural characteristics (Nagi and Mahns, 2010, 2013; Djouhri, 2016). The hedonic feelings felt with the fingertips still remains to be explained. Recent studies have identified MrgprB4 neurons in mouse as being involved in the pleasure felt during massage. In the experience realized, the MrgprB4 neuron was only activated the mouse was stroked with a brush. Results suggest that CT fibers with MRGPRB4 have distinct sensitivity to other anatomically similar CT neurons with receptors (MRGPRD) sensitive to unpleasant stimulation (Vrontou et al., 2013). This new knowledge could partly explain the gentle touch felt by the extremity of the finger.

A study realized on healthy subjects by Functional magnetic resonance images (fMRI) show that soft brush stroking activates the somatosensory areas S1 and S2, as well as insular cortex, notably the posterior part of the contralateral insular cortex (Olausson et al., 2002). S1 and S2 receive fibers Aβ projections and are known to play crucial roles in discriminative touch. When similar brushing stimuli are applied to GL, no activation is found in the somatosensory areas when the posterior insular region is activated (Olausson et al., 2010). The unmyelinated CT afferents, therefore, probably have excitatory projections mainly to emotion-related cortical systems such as the insular cortex (Vallbo et al., 1993; Vallbo et al., 1999). Other studies (Rolls et al., 2003) showed that the orbitofrontal cortex is involved in representing both positive and negative affect produced by touch, as it is the case with a stimulation through other sensory modalities (Rolls, 2000).

The emotional response triggered by a stimulus can be studied according to its three components: the expressive component (or behavioral response) measured by electromyogram of facial muscles, the cognitive component (feeling experienced) evaluated by self-assessment questionnaires, and the physiological component (or autonomic activity). Recent technologies allow now scientists to measure the emotions more accurately. For instance, in an array of pictures, those inducing fear (e.g., snakes) are located faster than the others (Öhman et al., 2001). Moreover, between 60 and 100 milliseconds after displaying an unpleasant stimulus, those inducing fear produce larger amplitude of visual evoked potentials than the others (Stolarova et al., 2006).

The first data showing a clear correlation between pupil diameter change and emotion were published in the sixties (Hess and Polt, 1960). Later, Bradley et al. (2008) have used emotional images to study their effect on the pupillary response. They divided pictures of the International Affective Picture System (IAPS; Lang et al., 2005) into three equal parts being considered, respectively, as pleasant, neutral and unpleasant. The results showed that the diameter of the pupil is significantly affected by the emotional pictures: the pupil becomes more dilated with emotional pictures, whatever its valence (i.e., pleasant or unpleasant). Additionally, Bradley et al. (2008) reported that the pupil diameter changes are correlated with changes in the conductivity of the skin, but not with the heart rate. This is consistent with the idea that the pupil diameter reflects the activity of the sympathetic nervous system. Recently, other studies suggest that non-visual emotions can also increase the diameter of pupils. This has been observed with participants listening to emotional sounds (Partala and Surakka, 2003; Babiker et al., 2013) or testing emotional products (Lemercier et al., 2014). In neuropsychology domain, by studying the effect of emotional stimuli on pupil diameter of people with Parkinson’s disease, Dietz et al. (2011) have found a normal sympathetic excitation to affective stimuli (indexed by pupil diameter), but no oculomotor differences.

The pupillary response reflects the activity of the autonomic nervous response, especially the parasympathetic and sympathetic systems (Charier et al., 2017). The parasympathetic system innervates the sphincter pupillae and controls the pupil constriction, whereas the sympathetic nervous system causes the excitation of the dilator pupillae (Beatty and Lucero-Wagoner, 2000). Initially, the major role of these muscles is to adjust the amount of light allowed to enter the eye (Ellis, 1981). Recently, a review of 134 studies (Kreibig, 2010) has shown that many theories agree on the relationship between emotion and the organization of autonomic nervous system activity. In this way, many authors propose to adopt an observation of the results according to the type of emotion examined. This is consistent with the results of Bradley et al. (2008). An increase of pupil diameter and sweating is observed in pleasant and unpleasant conditions, whereas the heart rate slows down for negative stimuli and increases with neutral and pleasurable condition. Other researchers have studied heart rate variability in different emotional states (Onorati et al., 2013; Park and Thayer, 2014).

It is now well-known that touch involves a sensory and emotional integration (Chaplan et al., 1994; Armaied et al., 2005; Rolls and Kesner, 2006; Spapé et al., 2015; Schirmer and Adolphs, 2017). Nevertheless, few studies have examined the activity of the autonomic nervous system during an exploration by touching a surface. Thus, the present study proposes to measure the emotional touch of different surfaces with explicit (verbal rating scale) and implicit (pupillometry) measures. We expect that the tactile exploration of an emotional material will increase the activity of the dilator muscle. Therefore, pupil diameter is supposed to increase with material surfaces with high emotional Intensity, regardless their hedonic valence.

## Experimental Procedure

### Participants and Materials

A preliminary study was conducted to select test materials, within a library containing 50 different samples (*wood, fabrics, furs, skins, metals, plastics, slimes, sandpaper)*. This library was created according to the recommendations on the representation of the pleasant touch specified in the literature (Greenspan and McGillis, 1991; Francis et al., 1999; Rolls et al., 2003; Thieulin, 2017). Six participants were asked to estimate the emotional valence and intensity of the surfaces, and two set of six materials were selected. The set 1 contains six surfaces obtained by 3D printing of six different neutral materials. Three of them are made of 100% polylactic material (green, pink, turquoise), and the others contain 50% of wood, copper and titanium powder, respectively. The measured roughness coefficients of these samples range from 0.255 to 0.417. The set 2 is made of six emotional surfaces. Two of them are considered as pleasant (very soft velvet and artificial fur). The three others are different grades of sandpaper, with an increasing roughness, i.e., N1 (slightly rough), N2 (moderate rough) and N3 (very rough). They are considered as unpleasant.

A panel of twenty-five students, 12 men and 13 women aged from 18 to 27, was selected. According to the “Edinburg” test which allows calculating the coefficient of laterality (Q.L.) on the basis of the examination of 12 gestural tasks (Oldfield, 1971), the panel was found to be right-handed, except for a female subject. According to the “Von Frey” test, which measures the changes in tactile sensitivity by using five calibrated filaments (Von Frey, 1896), all participants have a normal tactile perception. Finally, the emotional state of the participants before the test was estimated by using the questionnaire of the “HAD – Hospital Anxiety and Depression scale” (Zigmond and Snaith, 1983). Four subjects presented a score above the pathological limit. Therefore, the HAD scores were introduced as covariate in our analyses. Since these data were found to induce no measurements bias, the twenty-five students were kept for the test.

### Procedure

Emotion outcome was assessed (i) via a self-report judgment (explicit measure), to collect information on intensity and valence (Russell, 1980), and (ii) via measurements of the autonomous nervous system using a pupillometer. Since the works of Osgood (1969) and Russell (1980), stimuli can be characterized by two dimensions, valence and intensity. Valence refers to the hedonic nature of information on a continuum ranging from “positive, pleasant” to “negative, unpleasant” while the intensity corresponds to the level of physiological activation provoked by emotional information on a continuum ranging from “calm” to “excited”. Many authors (Bradley et al., 2008) have shown that pupillary responses might serve as a reliable index of emotional intensity and other works have shown the interest of collecting the emotional value of a material (Bertheaux et al., 2018). This physiological response is considered as more automatic and implicit than explicit, verbalized self-report measures. Pupil diameter is modulated by emotional intensity (linked to somaesthetic cues). Studies comparing and correlating emotional responses about their peripheral physiological mechanisms and self-report judgment, are limited in number.

The participants were sitting on a comfortable chair, the arms resting on a table to limit the movement of the body, in an artificially lighted room with a small opening on the outside. Luminance measurements gave values ranging from 240 to 250 Lux. A 850 × 850 mm white wooden tray with a target point in its center was placed at 40 cm in front of the participants. This experimental equipment was used to focus the gaze toward a target point to facilitate the measurement of the pupil and to avoid any distractions. To perform a blind experiment, the materials were placed in an experiment box with 6 materials in 6 housing spaced 25 mm. The box dimensions were 700 mm long, 140 mm high and 200 mm deep. The samples were 70 × 70 × 5 mm to allow exploration of the surface with 3 fingers per tangential and circular touch. Randomly ordered set 1 and 2 were presented successively to the participants.

**FIGURE 1:**
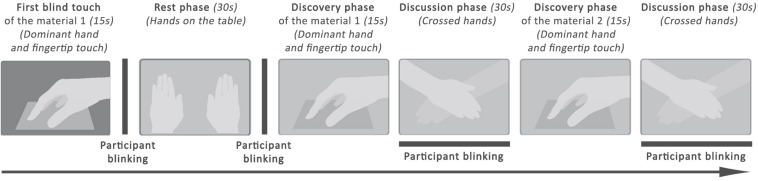
Experimental procedure applied on each set.

During the test, an experimenter was placed behind the subject so that the participant was not tempted to look at him during the answers. He described each step to the participant who executed the experiment. A second experimenter, located beside the participant, registered markers in order to sequence each measurement step. Figure 1 gives the different steps of the procedure. The first step consisted in a blind touch during 15 s of a randomly chosen material of the set. This step erased the “surprise” effect, and the associated data are not processed. It was followed by a recorded “Rest phase” of 30 s, which was used to define the initial pupil diameter of the participant d_0_. Then, participants silently touched each material during 15 s with the dominant hand. After this touching period, the experimenter asked them about his feelings and noted their answers during 30 s. Valence was assessed using a scale from –4 (very unpleasant) to +4 (very pleasant), and intensity is ranged from 0 (very calm) to 5 (very excited).

The scales used for valence and intensity are adapted from the SAM scale (Self Assesment Mankini), which proposes to measure the emotion on three dimensions: pleasure (valence), excitation (intensity) and dominance (Bradley and Lang, 1994). During the “Discussion Phase”, the participant said whether the sample was pleasant (yes or no) and indicated his feeling on the valence scale (−4 to 4). Then, the participant said whether he has felt an emotion (yes or no), and quantified it with the intensity scale (0 to 5). A written example is shown in Table 1.

**TABLE 1:** Sample of survey used in the subject assessment.

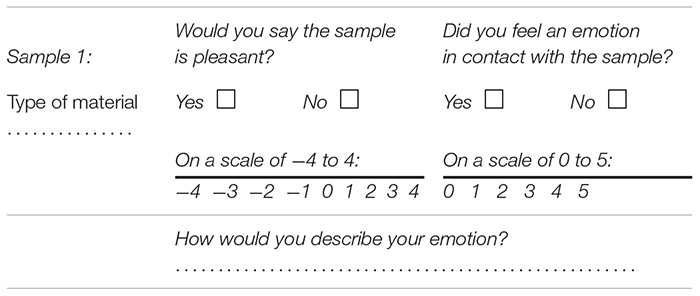

Pupil diameter measurements were performed at 60 Hz with an ISCAN-ETL-100H pupilometer device connected to an infrared camera. The size of the pupil in pixels is detected in the vertical and horizontal directions, and the mean value at time t gives de pupil diameter d(t).

## Results and Discussion

### Questionnaire

**FIGURE 2:**
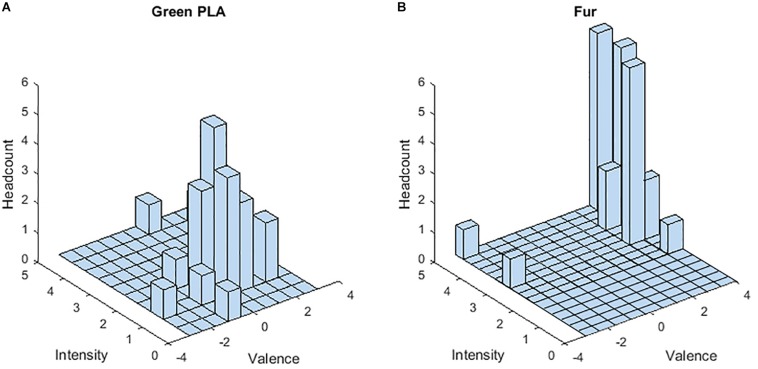
Emotional values (valence and Intensity) of two samples. Histograms of Green PLA **(A)** and Fur **(B)** as a function of Intensity and Valence.

Figure 2 gives the histograms of emotional values collected by the questionnaire for two samples, “Green polylactic (PLA)” (Figure 2A) and “synthetic fur” (Figure 2B). In Figure 2A the values are clearly distributed around a mean value of 0.6 (intensity) and 1.76 (valence). The emotional value of this sample is considered as “neutral”. In Figure 2B, the intensity values are highly positive, between 3 and 4, with a mean value of 3.12, and the valence is mainly positive, with a relatively high mean value of 3.84. The emotional value of this sample is therefore considered as “pleasant”. However, it can be observed in Figure 2B that two participants have considered the synthetic fur as very unpleasant, with two ratings at −3. This can probably be explained by the cultural background of these two participants who raised in an environment with wild animals. Actually, the furry animals are often considered as pleasant. Often during our childhood, we experiment the soothing and relaxing effect of touch with a cat, a dog, a teddy bear … (Servais, 2007). It does not represent a danger. Nevertheless, the two students lived in a country where animals are dangerous, so that they did not consider the fur as pleasant as the other students.

Considering all the participants, the mean value of the verbalized valence was found to be 2.74 ± 1 for the two pleasant materials (very soft velvet and artificial fur), −0.41 ± 0.55 for the three unpleasant materials (sandpapers), and 0.77 ± 0.07 for the seven remaining neutral materials. A Friedman test on the valence ratings showed that these three conditions of emotion were significantly different (χ^2^ = 50, *p* ≤ 0.0001), and *a posteriori* Wilcoxon analysis showed significant differences between the three dimensions (*p* < 0.0001). Similarly, the mean intensity ratings for the pleasant, unpleasant and neutral touch stimulus across all subjects were, respectively, 3 ± 0.16, 2 ± 0.10, and 1.9 ± 0.17. A Friedman test on these intensity ratings showed that the three conditions of emotion were significantly different (χ^2^ = 48, *p* ≤ 0.0001). *A posteriori* Wilcoxon analysis showed significantly higher score for pleasant stimuli compared to neutral and unpleasant stimuli (*z* = 4.37, *p* ≤ 0.0001 and *z* = 4.37, *p* = 0.0001, respectively), and the pattern of results indicated higher mean intensity score for negative stimuli compared to neutral touch (*z* = 4.13, *p* = 0.0001). No specific gender differences appeared and the HAD score had no influence on the scales scoring.

### Pupil Diameter Change

A cleaning algorithm has been applied to all the recorded diameters to detect blinks, absence of measurements, and measures considered aberrant (pupil size too high). After detection, the algorithm uses a numeric interpolation and replaces this data with a corrected data bridge that covers the mapped interval (Schlomer et al., 2010). For each material and for the rest phase, the recorded pupil diameters were exploited for 2 s. This duration was defined according to the work of Bradley et al. (2008), which shows that the constriction of the pupil intervenes from 0.6 to 1.6 s after the presentation of an image (emotional stimuli).

The data processing was finally performed by calculating the relative evolution of the pupil diameter of each subject, for each material, during the discovery phase, as [d(t)-d_0_]/d_0_. The d_0_ value is obtained by averaging the pupil diameter values during the last 2 s of the rest phase for each subject. Participants observed two periods of Rest phase, one taken during the measurement of set 1 and the other during set 2. The d(t) value is the pupil diameter of the participant measured at time t during the first 2 s of the discovery phase.

**FIGURE 3:**
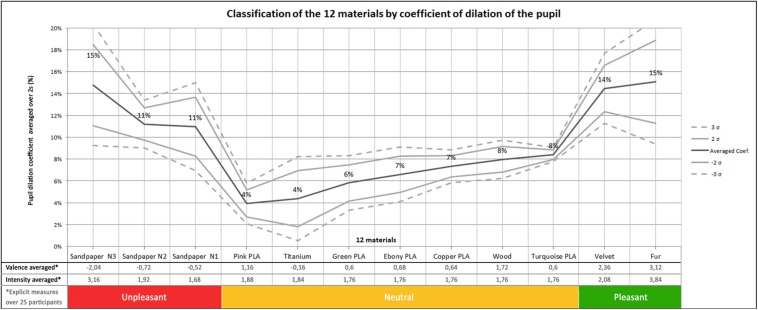
Classification of materials by coefficient of dilation of the pupil on 3 conditions: unpleasant, neutral, pleasant.

Figure 3 shows the time and participant averaged pupil dilatations for all materials, classified from unpleasant to pleasant, together with the mean verbalized valence and arousal. According to the Bienaymé-Tchebychev Inequality Test, in the case where the data do not follow a normal law, the probability that the random variable will be realized in interval ±2 σ is 75%, and becomes 89% in the range of ±3 σ. It can be observed in this figure that high emotional materials present larger pupil dilatations than neutral materials, but that pupil dilations for unpleasant and pleasant materials are very similar.

Figure 3 suggests that our statistical analyses can be performed with the following three classes of materials:

– Three Unpleasant: sandpapers from N1 to N3;
– Seven Neutral: all PLA, Titanium and Wood;
– Two Pleasant: Velvet and Fur.

We performed the Shapiro–Wilk test on this dataset. At 5% risk, our results showed that our data don’t follow a normal distribution. Therefore, all data were rank transformed (Conover and Iman, 1981) and submitted to an ANCOVA. The HAD score was used as covariate since previous studies showed that the level of mood can influence the processing of emotional materials (Mogg and Bradley, 1999; Mogg et al., 2000). We included in the present analyses the inter-subject factor “group” (male and female) and the within-subject factor “emotion” (pleasant, unpleasant and neutral). In addition, pairwise *post hoc* comparisons with the Bonferroni test and planned comparisons were performed when appropriate. The ANCOVA showed no effect of the HAD score on the overall results, but the “group” and emotion” factors were found to be significant.

**FIGURE 4:**
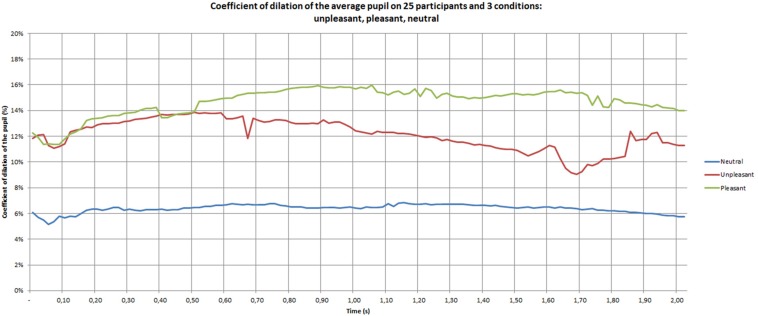
Pupil dilation coefficient of the participants during the discovery phase.

The effect of emotion is significant [*F*(2,24) = 10.87, *p* = 0.0004]. Figure 4 shows the pupil dilation coefficient of the participants during the discovery phase. These data are averaged over the three classes of materials: neutral, unpleasant, pleasant. The following observations can be made on this graph:

– The neutral materials lead to a lower pupil dilation than the pleasant [*F*(1,12) = 146.07, *p* = 0.0001] and unpleasant materials [*F*(1,12) = 11.99, *p* = 0.004];
– A constriction of the pupil is located around 0.05 s;
– After the constriction zone, the neutral curve is fairly constant in time, the pleasant curve increases during 0.5 s, and then stay roughly constant and the unpleasant curve stay constant during 0.5 s, before decreasing with greater variability on the end.

An observation conducted on the temporal data showed that the emotional conditions of pleasant and unpleasant materials become different from 0.50 to 2 s (*p* = 0.03).

The group factor (male and female) was found to also significant [*F*(2,24) = 3.42, *p* = 0.04]. The pupil is more dilated in women than in men under both pleasant and unpleasant conditions (*p* = 0.05 and 0.0001, respectively).

**FIGURE 5:**
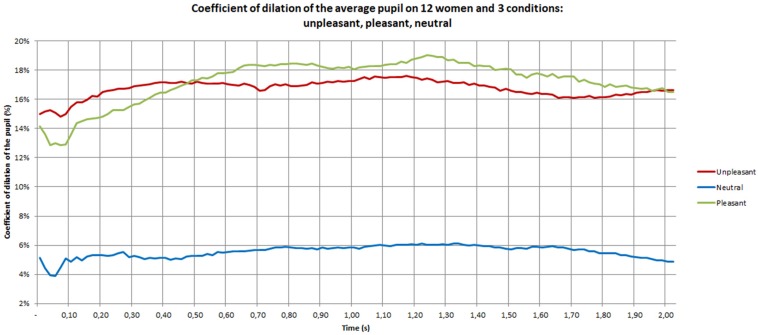
Pupil dilation coefficient of women during the discovery phase.

As shown in Figure 5, the pupil dilation coefficients of women are situated between 13 and 19% for the unpleasant and pleasant conditions, while they range between 4 and 6% for the neutral materials. The *post hoc* analysis showed a difference between unpleasant and pleasant (*p* = 0.01), unpleasant and neutral (*p* = 0.001) and pleasant and neutral (*p* < 0.0001) materials. The positive stimuli seemed to be the most emotional for women. In women, the curves showed that after 0.50 s the positive stimulus increased and the neutral and negative settled down.

**FIGURE 6:**
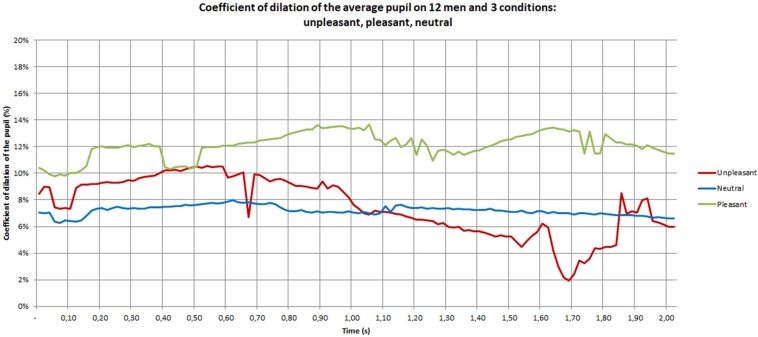
Pupil dilation coefficient of men during the discovery phase.

The pupil dilation coefficients of men are depicted in Figure 6. They range between 2 and 12% for the unpleasant and pleasant conditions, and between 6 and 8% for the neutral condition. Furthermore, the curve presents much more variation than for women. The *post hoc* analysis showed a difference between unpleasant and pleasant (*p* = 0.004), unpleasant and neutral (*p* = 0.009) and pleasant and neutral (*p* < 0.0001) materials. During the 2 s observed, the neutral curve is nearly constant. The pleasant stimulus shows large oscillation with a first peak at 1 s and a second peak at 1.70 s. The negative stimulus is located above the neutral and below the positive during the first second, and then a sharp decreased occurs that places the negative stimulus below neutral and pleasant conditions. Pleasant stimuli seem to be the most emotional for men.

**FIGURE 7:**
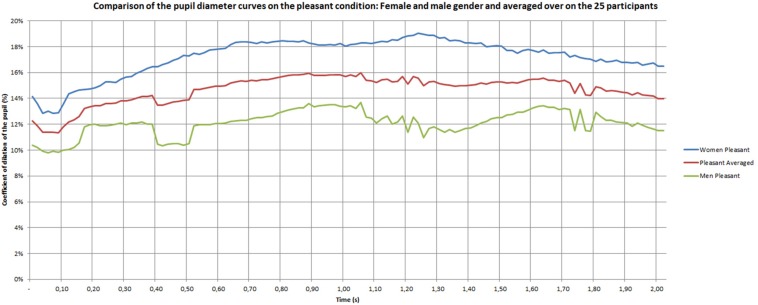
Pupil dilatation coefficient for pleasant materials.

**FIGURE 8:**
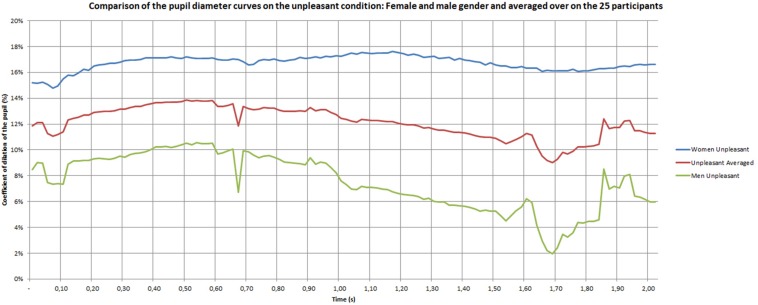
Pupil dilatation coefficient for unpleasant materials.

**FIGURE 9:**
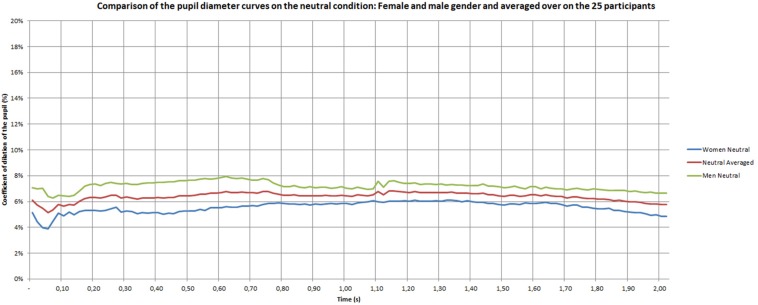
Pupil dilatation coefficient for neutral materials.

Figures 7–9 give the pupil dilation of men, women, and averaged, in the case of pleasant, unpleasant, and neutral materials. It can be observed in these figures that:

– for the pleasant materials: the pupil dilation is larger for women than for men, with similar time evolutions, but with similar curves slightly noisy in the men case (Figure 7);
– for unpleasant materials: the pupil dilation is greater for women than for men with different time evolutions: nearly constant for women, and decreasing after 0.70 s for men (Figure 8);
– for neutral materials: the pupil dilations are roughly similar for men and women (Figure 9).

**FIGURE 10:**
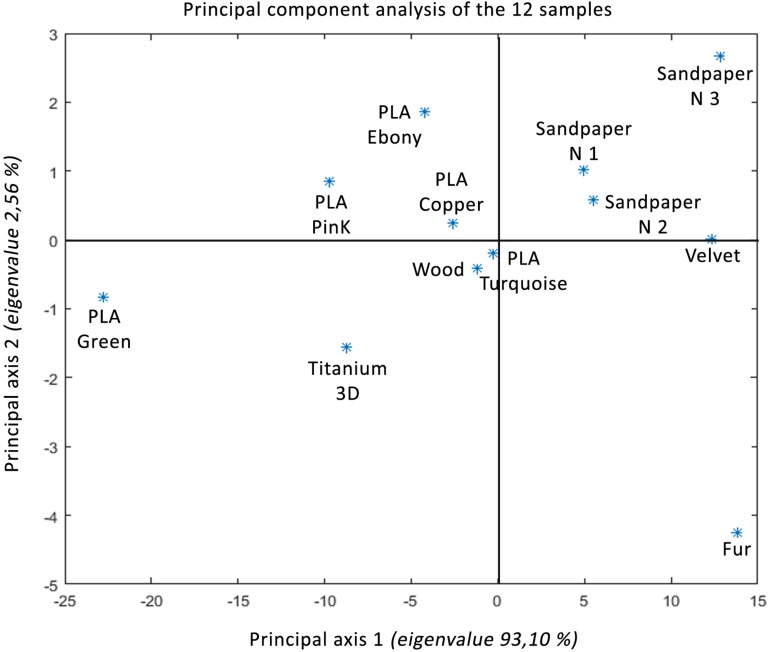
Principal component analyzis of the twelve samples.

In Figure 10, a Principal Component Analysis (PCA) classifies the materials according to the mean value of the pupil dilation. Principal component analysis is a method of eliminating bias. On the horizontal principal axis, which represents 93.10% of the values, the materials are clearly ordered according to their Intensity, from neutral to emotional. This ranking is close to the classification made by the participants using subjective measures. On the vertical axis, which represents 2.56% of the values, the ordering of the materials is more difficult to analyze.

## Discussion

The aim of this study was to evaluate pupillometry as a measurement technique for emotion. For this purpose, an experimental procedure has been developed, and the touch of different surfaces has been used as a stimulus. The results show clearly an increase in pupil diameter within the first 2 s of the touch. As observed by Bradley et al. (2008), the initial increase is followed at about 0.04 until to 0.10 s by a slow decrease. After, the averaged pupil dilation follows a similar type of curve whatever the emotional materials until a period of about 0.5 s. The general pattern of results indicate a larger pupil size dilation in response to both negative (unpleasant) and positive (pleasant) stimuli, when compared with the dilation induced by neutral stimuli. After this time period of 0.50 s, the emotional response takes different ways. The pupil diameter tends to decrease for negative stimuli, whereas it stays constant for positive stimuli.

Pupil diameter can be considered as a physiological marker of the autonomic nervous system. The size of the pupil is fixed by the relative activity of the two iris muscles, the sphincter and the dilator (Beatty and Lucero-Wagoner, 2000). While pupil constriction is maintained by parasympathetic activity, pupil dilation is essentially induced by the sympathetic pathway (Andreassi, 2000). Because the size of the pupil is modulated by the autonomic nervous system, our results suggest that this system reacts differently to emotional and to neutral stimuli. This pattern of results confirms many studies suggesting that emotional intensity during picture viewing, sound auditioning, or tasting, is associated with high pupil dilation (Partala and Surakka, 2003; Bradley et al., 2008; Kret et al., 2013; Henderson et al., 2014; Lemercier et al., 2014). Bradley et al. (2008) indicated that the dilation of pupil diameter covaried with other autonomic measures of intensity, such as the skin conductance, confirming that pupillary responses can be considered as a reliable index of emotional intensity. More recently, Laeng et al. (2016) showed that emotional music may reveal changes in the diameter pupil and that a neuromodulator role of the central norepinephrine system is involved in this phenomenon. To our best knowledge, nobody has undertaken any studies on the response of the autonomic nervous system to touch.

Some studies indicated that changes in pupil diameter specifically occur within the first few hundred milliseconds after stimulus onset, with responses peaking after 1 to 2 s (Loewenstein and Loewenfeld, 1962; Andreassi, 2000; Beatty and Lucero-Wagoner, 2000; Nieuwenhuis et al., 2011). Beyond 2 s of regulation, we can also suppose larger pupil diameter. For example, Bradley et al. (2008) showed differences when participants viewed emotional or neutral pictures beyond 2 s. Partala and Surakka (2003) observed this effect with auditory stimuli. Our results show a dynamic pattern during the 2 s time period. Initially, unpleasant touch does not induce any difference compared to pleasant touch, but a difference can be observed after 0.50 s. In the same way, Partala and Surakka (2003) results showed that no pupil dilatation was observed before 0.40 s and that the maximum pupil dilatation was observed after 2 to 3 s. In addition, they showed that the time duration of the pupil diameter were somewhat different for female and male subjects. The positive stimuli provoke the strongest pupil dilations for female subjects, whereas the negative stimuli provoked the strongest dilations for male subjects.

As Partala and Surakka (2003), the present study showed an effect of gender but in a different way. The strongest pupil dilations appeared for both emotional conditions with women. This is constant with the duration time. For men, the emotional conditions induced a higher pupil dilations compared to neutral stimuli at the beginning. Nevertheless, a strongest decrease for negative emotion appeared with the time period. The explanation of a higher sensitivity in female participants could be explained by the difference of the skin thickness. It exceeds 60 microns for men (between 20 and 30 years old) and is often below 50 microns for women (Kawabe et al., 1985). For example, Peters et al. (2009) indicate that women can perceive finer surface details than men, and that tactile perception is improved with decreasing finger size (Van Boven et al., 2000; Goldreich and Kanics, 2003, 2006). An explanation of this phenomenon of better perception in women comes from the high density of Merkel’s receptors and Meissner’s corpuscles due to the small size of their fingers (Dillon et al., 2001; Peters et al., 2009). Other differences were found between men and women, like variability of vibrotactile detection thresholds, the volume of the area of contact with the material and a higher density of ridges in the fingerprint (Gutiérrez-Redomero et al., 2011; Venkatesan et al., 2015).

In the pain studies, many authors showed that the threshold of nociception by pressure decreases with age, especially in men. In contrast, age seems to have no effect on thermal sensations (Pickering et al., 2002). Another study showed that the temporal sum of mechanically evoked pain is seen with higher scores in women than in men (Sarlani and Greenspan, 2002). Studies of pupil changes due to painful pressure stimuli showed that increasing pupil diameter was a highly significant indicator of pain intensity. In this domain, female subjects show greater dilation than men at high pressure levels (Ellermeier and Westphal, 1995). These data lead us to the conclusion that the pupil dilation seems differently influenced by pain or emotion following the gender.

From a cognitive approach, Bayer et al. (2011) indicated that pupillary responses are sensitive to both task load and emotional content. Kuchinke et al. (2007) showed that word frequency significantly affected pupil dilation. The pupil diameter appeared to increase for low frequency words certainly because this type of words induced a more important cognitive load. In addition, cultural aspects are important in the touch of materials. This was demonstrated by Fisher (2004) in a study which suggests that consumer perceptions of plastics are physical and emotional. This researcher considers that the properties of plastics are “humanized” by different industrial processes making this material harmless, sensual and familiar unlike the animal or natural properties of some materials who remind us to our natural environment that can arouse more excitement. The appreciation of these materials varies according to the context, the emotional charge produced by its visual and tactile properties (Gibson, 1977) or cultural aspects due to habits. For example, sandpaper is mainly used by men in construction; manufacturing and its surface appearance may seem more familiar to them. Therefore, pupil dilation may reflect the time course of cognitive and attentional mechanisms inherent in emotion processing. Therefore, we can imagine that the pupillometry informs us as an emotional intensity (Rolls et al., 2003; Bradley et al., 2008) and can allow to show a pattern of results, with increase and decrease regulation (Urry et al., 2009), as it is the case in our study.

There is some evidence that self-report emotional answers vary with certain physiological changes associated with emotion (Levenson, 1992). In our study, we show that the materials are subjectively classified in the same order as the classification of the coefficient of dilation observed for the 12 materials. Thus, the main axis 1 seems to correspond to the bodily excitation of the tactile stimulus (Intensity). Nevertheless, the ratings of intensity showed that negative and positive stimuli were experienced as differently arousing. Our pattern of results justifies the use of verbal response in complement with physiological data. On one side, unpleasant touch was assessed as significantly less arousing than pleasant touch. On the other side, the positive intensity effect manifested specifically later during the pupillary response compared to the negative intensity effect. In accordance with subjective ratings, increasing positive emotions led to the most prominent pupil size enlargements during 2 s. These results confirmed that pupil diameter was modulated by the level of emotional intensity. Results suggested that pupil responses reflected the time course of the intensity perceived. According to Gross (1998), emotion regulation refers to both implicit, explicit physiological, behavioral and cognitive processes. The inclusion of multiple measures of autonomic intensity with subjective and behavioral emotional ratings might help to understand effects reflecting changes in emotional Intensity and cognitive demand.

In sum, the present study opens new research avenues. Our data evidence that the pupillary response in accordance with subjective data might include distinct temporal components reflecting emotional and cognitive regulation. It would be interesting to develop pupil diameter variation as a computer signal input. For example, it has been possible to develop signal analysis methods to detect successfully emotion from electrical activity of facial muscles.

## Data Availability Statement

The datasets generated for this study are available on request to the corresponding author CBe, cyril.bertheaux@enise.fr.

## Ethics Statement

Ethical review and approval was not required for the study on human participants in accordance with the local legislation and institutional requirements. The patients/participants provided their written informed consent to participate in this study.

## Author Contributions

CBe is doing his thesis at Université de Lyon, ENISE, LTDS, UMR 5513 CNRS on the emotional impact of touch and more particularly in the tactile exploration of materials. This research work aims to determine the emotional value of materials. For this multidisciplinary thesis, a partnership with the laboratories SNA-EPI Laboratory, EA 4607 (DC, pupil and ANS specialist) and CMRR, neuropsychology of the CHU Saint-Étienne (CBo, Thesis guide, specialist in emotion and cognition) was established to provide knowledge in metrology of the autonomic nervous system and in cognition. The rest of the management is made up of engineers researchers (RF, Thesis Director and RT, Thesis Co-director) and statisticians (J-CR, Thesis guide) who have contributed their skills to the various protocols and results collected. All authors contributed to all the steps until the writing of this article. Since 2016, several test protocols, data organization, interpretation, correction, mathematical and statistical treatments have made it possible to propose these results and manuscript revision, read, and approved the submitted version.

## Conflict of Interest

The authors declare that the research was conducted in the absence of any commercial or financial relationships that could be construed as a potential conflict of interest.
